# Trypsin-Like Serine Proteases in *Lutzomyia longipalpis* – Expression, Activity and Possible Modulation by *Leishmania infantum chagasi*

**DOI:** 10.1371/journal.pone.0010697

**Published:** 2010-05-18

**Authors:** Erich Loza Telleria, Adriana Pereira Oliveira de Araújo, Nágila Francinete Secundino, Claudia Masini d'Avila-Levy, Yara Maria Traub-Csekö

**Affiliations:** 1 Laboratório de Biologia Molecular de Parasitas e Vetores, Instituto Oswaldo Cruz, Fiocruz, Rio de Janeiro, Rio de Janeiro, Brazil; 2 Laboratório de Entomologia Médica, Centro de Pesquisas René Rachou, Fiocruz, Belo Horizonte, Minas Gerais, Brazil; 3 Laboratório de Biologia Molecular e Doenças Endêmicas, Instituto Oswaldo Cruz, Fiocruz, Rio de Janeiro, Rio de Janeiro, Brazil; Louisiana State University, United States of America

## Abstract

**Background:**

Midgut enzymatic activity is one of the obstacles that *Leishmania* must surpass to succeed in establishing infection. Trypsins are abundant digestive enzymes in most insects. We have previously described two trypsin cDNAs of *L. longipalpis*: one (*Lltryp1*) with a bloodmeal induced transcription pattern, the other (*Lltryp2*) with a constitutive transcription pattern. We have now characterized the expression and activity of trypsin-like proteases of *Lutzomyia longipalpis*, the main vector of visceral leishmaniasis in Brazil.

**Methodology and Principal Findings:**

In order to study trypsin expression profiles we produced antibodies against peptides specific for Lltryp1 and Lltryp2. The anti-Lltryp1-peptide antibody revealed a band of 28 kDa between 6 and 48 hours. The anti-*Lltryp2* peptide antibody did not evidence any band. When proteinaceous substrates (gelatin, hemoglobin, casein or albumin) were co-polymerized in polyacrylamide gels, insect midguts obtained at 12 hours after feeding showed a unique proteolytic pattern for each substrate. All activity bands were strongly inhibited by TLCK, benzamidine and 4-amino-benzamidine, indicating that they are trypsin-like proteases. The trypsin-like activity was also measured *in vitro* at different time points after ingestion of blood or blood containing *Leishmania infantum chagasi*, using the chromogenic substrate BAρNA. *L. longipalpis* females fed on blood infected with *L. i. chagasi* had lower levels of trypsin activity after 12 and 48 hours than non-infected insects, suggesting that the parasite may have a role in this modulation.

**Conclusions and Significance:**

Trypsins are important and abundant digestive enzymes in *L. longipalpis*. Protein production and enzymatic activity followed previously identified gene expression of a blood modulated trypsin gene. A decrease of enzymatic activity upon the parasite infection, previously detected mostly in Old World vectors, was detected for the first time in the natural vector-parasite pair *L. longipalpis*-*L. i. chagasi*.

## Introduction

*Lutzomyia longipalpis* is the principal vector of *Leishmania infantum chagasi* which causes visceral leishmaniasis in Brazil [1], [2]. When the infected hematophagous female feeds on the vertebrate host, *Leishmania* parasites are delivered through the proboscis in the feeding site [3]. To digest the ingested blood, the insect secretes proteases in the midgut lumen [4]. Among these, serine proteases such as trypsins are the most abundant digestive enzymes within the midgut of blood-sucking insects [5], [6]. Trypsins have been extensively studied in mosquitoes of major public health importance, such as *Anopheles* spp. and *Aedes* spp.. Besides their role in blood digestion, these proteases have been implicated in the establishment of infection of pathogens in their respective insect vector. For example, a chitinase of *Plasmodium gallinaceum*, necessary for ookinete escape from the peritrophic matrix is activated by an *Aedes aegypti* midgut trypsin [7].

In sandflies, sequencing of ESTs has identified numerous digestive enzymes transcripts in infected and blood-fed *Phlebotomus papatasi and L. longipalpis* [8]–[12]. Interestingly, in both species there seems to be a modulation of trypsin genes expression related to *Leishmania* infection [9]–[11].

Earlier studies found the survival of *Leishmania* to proteolytic attack in the sand fly midgut to be one of the crucial steps during the parasite development within the vector [13], [14]. Insect-parasite specificity has also been associated with the expression of serine proteases. Borovsky and Schlein [15] suggested that trypsin-like activities in the midgut of *P. papatasi* prevented successful infection of this vector by *Leishmania donovani*, while modulation of these activities by *Leishmania major* allowed its survival in this host. Additionally, elevated protease activity at the end of *Phlebotomus langeroni* digestion process prevented *L. major* from establishing infection [16]. Recent experiments in *L. longipalpis* infected with *Leishmania mexicana* showed a decreased trypsin activity in infected insects [17].

We have previously described the transcription pattern of two *L. longipalpis* trypsin genes, *Lltryp1* and *Lltryp2* [18]. In the present report, we investigated the protein expression and activity of trypsin-like proteases during blood digestion and inferred a possible role for these enzymes on infection of *L. longipalpis* by *L. i. chagasi*.

## Materials and Methods

### Ethics Statement

All the animal procedures were approved by the FIOCRUZ bioethics committee (CEUA - protocol number P0-116-02).

### Chemicals

Trypsin substrate BAρNA, protein substrates, and protease inhibitors were purchased from SIGMA-ALDRICH (St. Louis, MO, USA). Freund's complete and incomplete adjuvants were purchased from GIBCO (Gibco BRL – Life Technologies, Grand Island, NY, USA). Horseradish peroxydase (HRP)-labeled goat anti-rabbit IgG was purchased from PIERCE (PIERCE, Rockford, IL, USA) and bovine trypsin from Amersham (Amersham Biosciences – GE Healthcare, Piscataway, NJ, USA). All other chemicals were analytical grade or superior.

### Sand flies

*L. longipalpis* were captured at Gruta da Lapinha, Minas Gerais, Brazil, and maintained in our insectary at Instituto Oswaldo Cruz, FIOCRUZ. Capture, maintenance and colonization in laboratory conditions were performed according to Brazil and Brazil [19]. Insects were fed on 30% sucrose solution *ad libitum*, and blood fed directly on an anesthetized male hamster when needed. F1 sand flies were used in all experiments. Experiments were performed using 3 pools of 10 insect midguts collected at 6, 12, 24, 48, and 72 h after blood feeding (ABF). Sugar fed insects (0 h) were used as control. Samples were frozen in solid carbon dioxide immediately after dissection, and kept at −70°C until use.

### Artificial blood-feeding and infection

Three-day-old female sand flies were fed through chick skin membrane on hamster blood containing 10^7^ promastigotes/ml from exponential growth phase culture of *L. i. chagasi* and flies were dissected at days 3 and 7 post-infection to confirm the presence of parasites [20]. The control group was artificially fed on hamster blood through chick skin membrane.

### Sand fly midgut extracts and protein quantification

For sample extraction, midguts were ground in 125 mM Tris-HCl pH 6.8 and 0.1% sodium dodecyl sulfate (SDS), frozen and thawed 3 times, centrifuged at 14.000 *g* for 30 minutes at 4°C, and the supernatant stored at −70°C until use. The extracts intended for enzymatic assays were prepared as described above, but were ground in 10 mM Tris-HCl, pH 8.0. Protein quantification was performed by the Bradford method using bovine serum albumin (BSA) as standard [21].

### *Leishmania infantum chagasi*

*L. i. chagasi* (strain MHOM/BR/1974/PP75) were obtained from the Instituto Oswaldo Cruz *Leishmania* Culture Collection. Promastigotes were maintained by weekly transfers in M199 medium, pH 7.0, supplemented with 10% fetal bovine serum.

### Synthetic trypsin peptides

**Figure 1:**
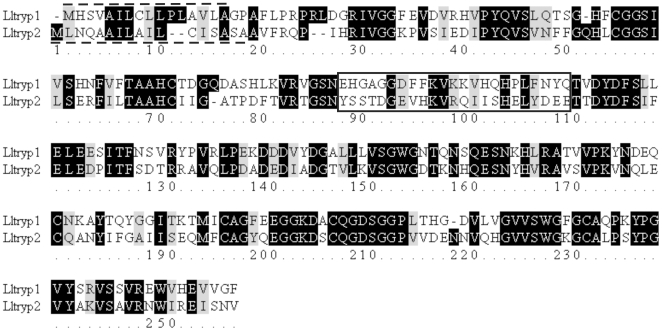
Alignment of deduced amino acid sequences of *L. longipalpis* trypsins (Lltryp1 and Lltryp2). Conserved residues are shown as white letter on black background. Non-conserved residues are shown as black letters on a white background. Black letters on gray background indicate conserved substitutions. The predicted secretory signal peptides for Lltryp1 (residues 2–17) and Lltryp2 (residues 1–19) are indicated by a traced line. Lltryp1 and Lltryp2-specific peptides used for immunization procedures are boxed (residues 89 to 111).

The trypsin peptides were designed to correspond to amino acid sequences that displayed low similarity between Lltryp1 and Lltryp2 (Figure 1) [18] in areas that were predicted to be highly immunogenic (http://www.bioinformatics.org/JaMBW/3/1/7/) using the Hopp and Woods algorithm [22]. Peptides were synthesized by Bio-synthesis Inc. (Lewisville, Texas, USA).

### Immunization

Lltryp1-peptide or Lltryp2-peptide (1 mg) were inoculated into 45 days old New Zealand male rabbits with Freund's complete adjuvant, with two subsequent boosts using incomplete Freund's adjuvant. Serum titration was carried out by enzyme-linked immunosorbent assay (ELISA), using the corresponding antigens, and revealed with horseradish peroxydase (HRP)-labeled goat anti-rabbit IgG as the secondary antibody.

### Western blot

Samples corresponding to 2 insect midguts were separated by 12% SDS-PAGE [23] under constant 100 V and transferred to nitrocellulose membranes during 1 hour at 4°C, using the same voltage. Membranes were blocked with 5% low-fat dried milk in Tris buffered saline (TBS) supplemented with 0.1% Tween-20. Membranes were washed three times with TBS and incubated for 1 hour with anti-Lltryp1-peptide or anti-Lltryp2-peptide sera at 1∶1,000 or 1∶100 dilution, respectively. HRP-conjugated goat anti-rabbit IgG at a 1∶40,000 dilution was used as secondary antibody. The relative molecular mass of the reactive polypeptides was calculated by comparison with the mobility of molecular mass standards, using ImageJ 1.42q software (NIH, USA).

### Zymography

Soluble protein samples were separated by 12% SDS-PAGE co-polymerized with protein substrates (gelatin, casein, hemoglobin and albumin) at 0.1% final concentration under non-reducing conditions [24]. Samples were loaded in the gel in non reducing buffer (125 mM Tris pH 6.8, 4% SDS, 20% glycerol and 0.002% bromphenol blue). Electrophoresis was carried out under constant voltage (100 V) at 4°C. The gels were washed in 2.5% Triton X-100 at 4°C under agitation during 1 hour to remove SDS. To test for best conditions, proteolysis of copolymerized proteins was performed by incubating the gel in different buffers: 0.1 M citrate (pH 4), or 0.1 M sodium phosphate (pH 6 and 8), or 0.2 M glycine (pH 10 and 12) for 18 hours at 37°C. All further zymography experiments were performed at pH 8. The proteolytic classes were determined by incubation of the gels at pH 8 buffer supplemented with the following specific protease inhibitors: *trans*-epoxysuccinyl L-leucylamido-(4-guanidino) butane (E-64) (10 µM) for cysteine-peptidases, pepstatin A (1 µM) for aspartic-peptidases, 1,10-phenanthroline (10 mM) for metallo-peptidases, phenyl-methyl sulfonyl-fluoride (PMSF) (1 mM) for serine-peptidases, tosyl phenylalanyl chloromethyl ketone (TPCK) (100 µM) for chymotrypsins, tosyl-lysyl-chloromethylketone (TLCK) (100 µM), benzamidine (1 mM), and 4-aminobenzamidine (1 mM) for trypsins. The gels were stained for 1 hour with 0.1% Coomassie blue R-250 in methanol:acetic acid:water (30∶10∶60) and destained in the same solvent.

### Enzymatic assay

The chromogenic trypsin substrate *N*α-Benzoyl-DL-arginine-ρ-nitroanilide (BAρNA) was used to quantify trypsin-like activity in midgut samples. A 25 mM stock solution of BAρNA was prepared in dimethyl sulfoxide (DMSO). The reaction was started by the addition of the equivalent to 0.5 insect midguts, in a total volume of 200 µL of 10 mM Tris-HCl pH 8.0, 4% dimethylformamide (DMF), and BAρNA substrate at 2 mM final concentration. This buffer was used as blank control and bovine trypsin (50 µg) was used as positive control. Cleavage of the chromogenic substrate was monitored continuously for 1 hour at 25°C in an automatic spectrophotometer for microtiter plates SpectraMax plus 384 (Molecular Devices, Sunnyvale, CA, USA) set to 410 nm [25], [26]. Optical density values were converted to enzymatic activity units based on ρ-nitroaniline standard curve [25], where one unit (1 U) of specific trypsin acivity is the amount of active enzyme which catalyses the formation of 1 µmol of ρ-nitroanilide per minute. Results are shown as the mean ± standard deviation of enzymatic units measured in three independent experiments in ten replicates each. The data were analyzed statistically by the Student's t-test using EPI–INFO 6.04 (Database and Statistics Program for Public Health) computer software.

## Results

### Trypsin detection by western blot

**Figure 2:**
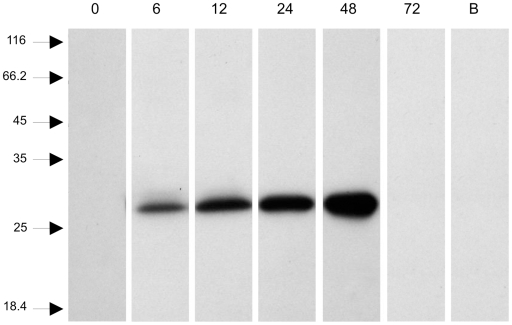
Western blot performed with anti-Lltryp1-peptide antibody. **0**: non-fed female; **6** to **72**: females obtained at 6, 12, 24, 48 and 72 hours ABF; **B**: 2 µL of hamster blood. Samples corresponding to 2 insect midguts were loaded in lanes **0** to **72**. Molecular mass standard values (kDa) are indicated by numbers and arrows on the left side of the figure.

Trypsin expression in *L. longipalpis* was analyzed by western blot (Figure 2). The anti-Lltryp1-peptide antibody revealed a band of approximately 28 kDa in midgut samples that increased in intensity from 6, 12, 24 to 48 h ABF. In samples obtained at 0 and 72 h ABF, and hamster blood, no band was detected (Figure 2). The anti-Lltryp2-peptide antibody revealed no band (data not shown). Western blots prepared from midguts of insects fed on *Leishmania* infected and non-infected blood showed a similar pattern of immunoreactivity with the anti-Lltryp1-peptide antibody (data not shown).

### Identification and characterization of peptidases by zymography

**Figure 3:**
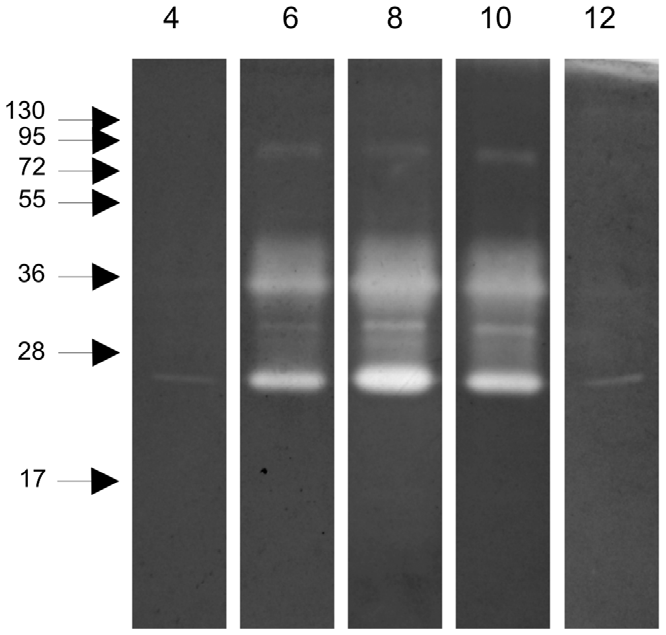
Zymography performed with gelatin co-polymerized gel, incubated in different pH defined buffers. **4** to **12**: pH of buffers. Samples corresponding to 1/50 of one insect midgut obtained at 12 h ABF were loaded in lanes. Molecular mass standard values (kDa) are indicated by numbers and arrows on the left side of the figure.

**Figure 4:**
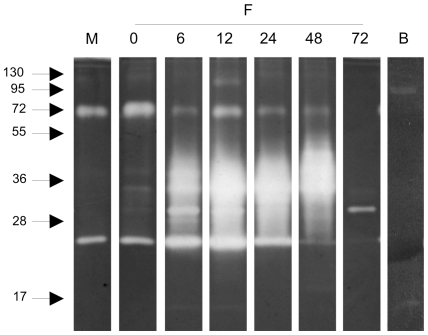
Zymography performed with gelatin co-polymerized gel incubated at pH 8 with samples obtained at different times ABF. M: males, **F**: females; **0**: sugar-fed females; **6** to **72**: females obtained at 6, 12, 24, 48 and 72 hours ABF; **B**: 2 µL of hamster blood. Samples corresponding to 1/50 of 1 insect midgut were loaded in lanes **M** and **0** to **72**. A control sample contained 1/50 of 2 µL of hamster blood. Molecular mass standard values (kDa) are indicated by numbers and arrows on the left side of the figure.

The proteolytic activity in the midgut was explored by zymography. Examination of the pH-sensitivity of the proteolytic activity using midgut samples collected at 12 h ABF and separated in polyacrylamide gels co-polymerized with protein substrate, revealed a pH optimum of 8, while no qualitative differences in proteolytic activity was detected at the various pH values tested (Figure 3). Additional midgut samples collected at different times ABF separated in polyacrylamide gels co-polymerized with gelatin (Figure 4) demonstrated proteolytic bands ranging from 17 to130 kDa when the gels were incubated under conditions to allow protein digestion. Hamster blood produced 2 weak bands with 17 and 95 kDa. Male and sugar fed female midgut samples showed 2 bands with 25 and 72 kDa that were also detected in blood fed female midgut samples. Confluent bands ranging from 25 to 55 kDa were detected between 6 and 48 h ABF. At 72 h ABF, the band profile changed dramatically with the loss of most of the enzyme activities detected up to 48 h ABF, revealing a discrete band of approximately 30 kDa.

**Figure 5:**
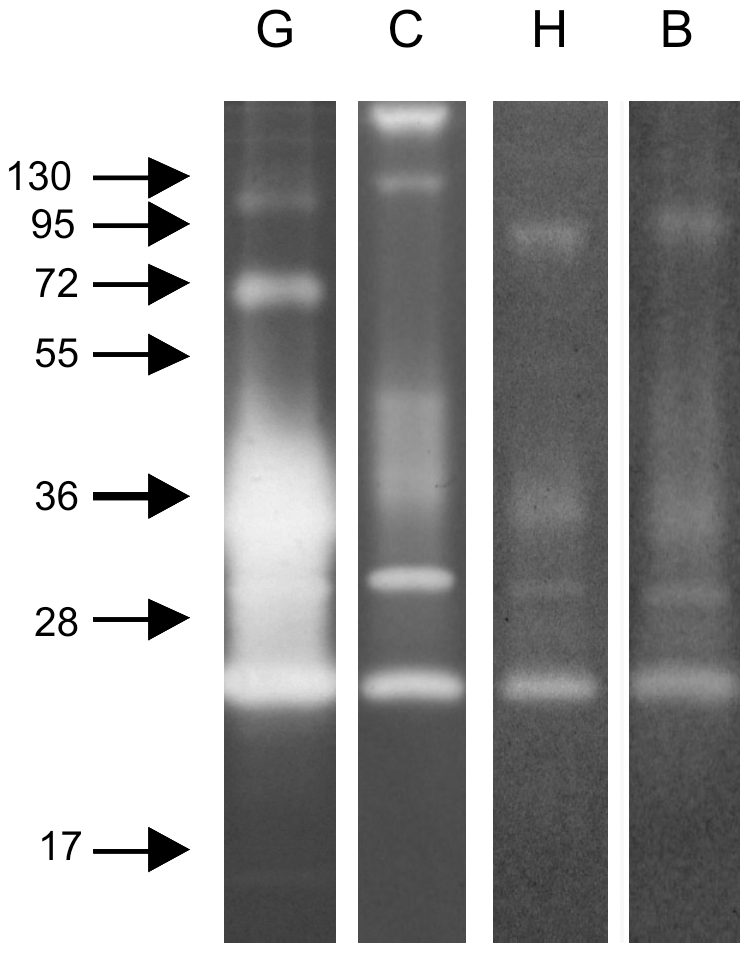
Zymography performed with gels co-polymerized incubated at pH 8 with different protein substrates. **G**: gelatin; **C**: casein; **H**: hemoglobin; **B**: BSA. Samples corresponding to 1/50 of 1 insect midgut obtained at 12 h ABF were loaded in lanes **G** and **C**, and 1/10 of 1 insect midgut obtained at 12 h ABF in lanes **H** and **B**. Molecular mass standard values (kDa) are indicated by numbers and arrows on the left side of the figure.

We also explored the ability of these enzymes to degrade distinct protein substrates. All substrates (gelatin, casein, hemoglobin and BSA) were degraded by midgut peptidases, with gelatin being most readily hydrolyzed under these conditions. Interestingly, casein co-polymerized gels revealed two additional strongly active bands at approximately 130 and 250 kDa, but did not demonstrate activity at molecular masses of 72 and 95 kDa. The enzymes ranging from 25 to 55 kDa were able to digest all the substrates employed (Figure 5).

### Protease class identification of zymography bands

**Figure 6:**
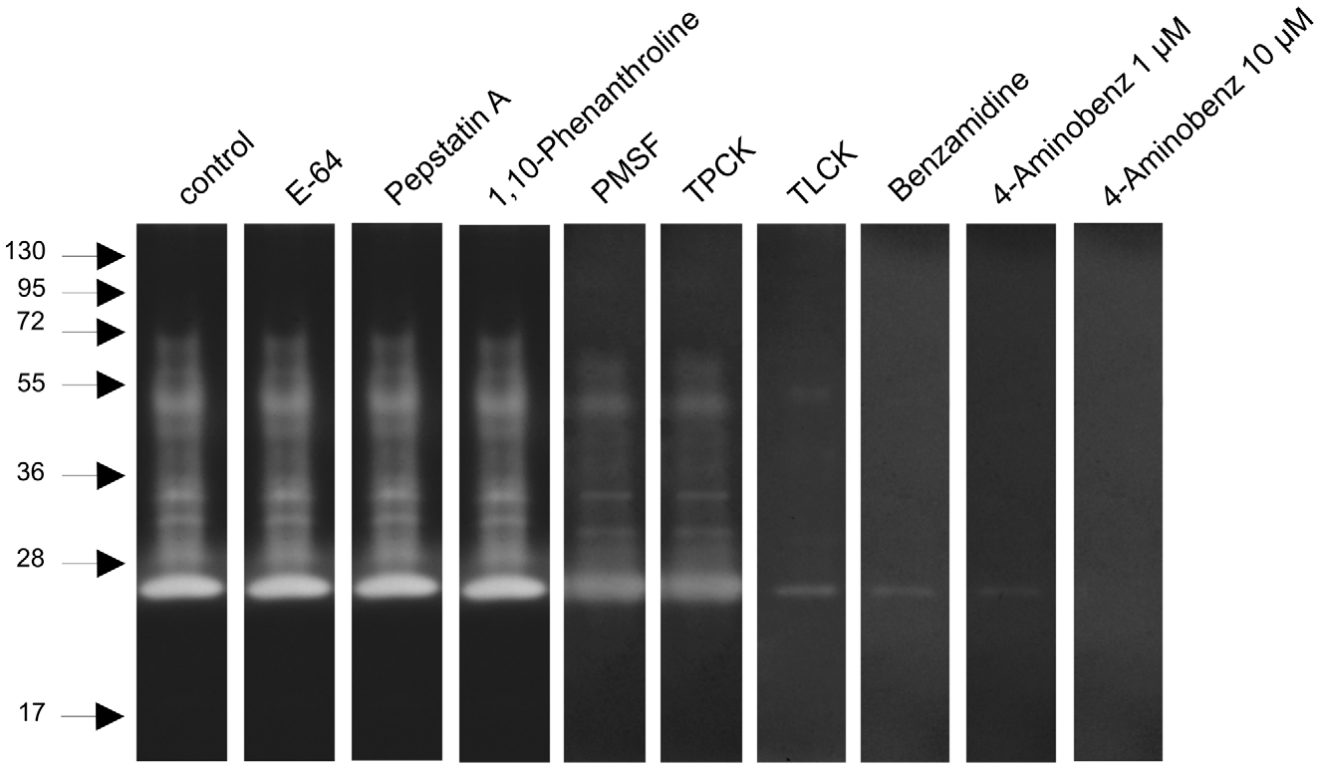
Zymography performed with gelatin co-polymerized gel, incubated at pH 8 with different protease inhibitors, as indicated. Samples corresponding to 1/50 of 1 insect midgut obtained at 12 h ABF were loaded in the gel. Molecular mass standard values (kDa) are indicated by numbers and arrows on the left side of the figure.

In order to better characterize the enzymes detected by zymography, a gel containing gelatin was incubated with specific inhibitors (Figure 6). Inhibition was determined by the absence, or substantial decrease, of proteolysis activities after incubation, which were assigned to the enzyme class of the correspondent inhibitor specificity. While cysteine-, aspartic- and metallo-protease inhibitors did not inhibit in-gel proteolysis, serine-protease (PMSF) and chymotrypsin (TPCK) inhibitors partially reduced proteolysis in all the identified degradation haloes. Trypsin inhibitors (TLCK, benzamidine and 4-amino-benzamidine) dramatically reduced all bands, and the 25 kDa band was only completely inhibited by high concentrations of the 4-aminobenzamidine inhibitor (Figure 6).

### Trypsin Enzymatic Assay

**Figure 7:**
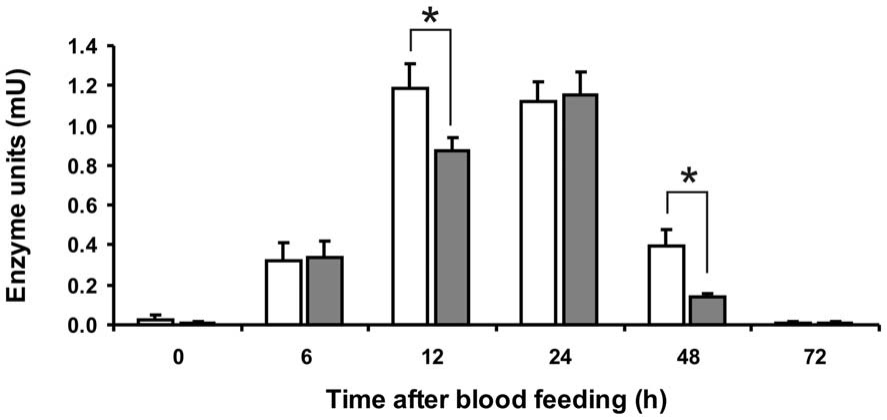
Enzymatic assay performed in solution with trypsin synthetic substrate BAρNA. Horizontal axis indicates insect midgut samples obtained at different hours ABF. Vertical axis indicate enzyme units detected per midgut. **0**: sugar-fed females; **6** to **72**: females obtained at 6, 12, 24, 48 and 72 hours ABF. White bars indicate samples obtained from non infected insect midguts. Gray bars indicate samples obtained from infected insect midguts. Samples corresponding to half of one midgut were used in each assay. Statistically significant difference (*P*<0.05) is marked with an asterisk.

Midgut samples were tested for trypsin-like activity in solution assays using a specific substrate. Activity corresponding to one adult midgut was measured (Figure 7). The proteolytic activity reached its peak at 12 h ABF, stayed almost unaltered until 24 h, and then decreased, returning to the basal levels after 72 h ABF. The activity peak of infected insect midguts was reached 24 h ABF and considerably lower activity was detected at 12 h and 48 h ABF when compared to uninfected samples. At 12 and 48 h ABF this difference was statistically significant (*P*<0.05).

## Discussion

Trypsins are the main digestive enzymes present in blood sucking insects and were shown to have a role in vector competence [27]. Trypsin transcription, expression and activity have been investigated in various insect vectors of medical interest. In *Anopheles gambiae*, among seven trypsin genes identified, five are constitutive (*Antryp3*, *Antryp4*, *Antryp5*, *Antryp6* and *Antryp7*), while two are blood meal-induced (*Antryp1* and *Antryp2*) [28], [29]. In *Anopheles stephensi*, constitutive and blood induced trypsins were identified by testing the enzymatic activity of midgut extracts on synthetic substrates, and by western blot analysis [30]–[32]. In *Aedes aegypti*, the early-trypsin activity was detected immediately after blood feeding (ABF) and late-trypsins reached a peak of activity one day ABF [33]–[35].

Phlebotomine trypsins have also been investigated. In *P. papatasi* three trypsin genes, *Pptryp1*, *Pptryp2* and *Pptryp3* are transcribed in non-fed females, while *Pptryp4* is transcribed after blood ingestion [36]. In *L. longipalpis*, we showed the transcription pattern of two trypsin genes similar to the ones identified by Dillon *et al*. [8]. One (*Lltryp2* - EF011107) had a constitutive transcription pattern, only marginally reduced when blood ingestion occurred, while the other (*Lltryp1* - EF011106) was induced by blood intake, with a peak of transcription at 12 h to 24 h ABF [18].

In the present work, antibodies raised against a specific Lltryp1 peptide showed a blood induced profile while the antibody raised against a specific Lltryp2 peptide failed to show any bands. The failure to detect Lltryp2 may be due to the low levels of this enzyme in the midgut. The *L. longipalpis* blood induced trypsin showed an expression pattern similar to the blood induced *A. gambiae* Antryp2 protein [29]. On the other hand, the anopheline trypsin Antryp4, which is transcribed constitutively, was detected by western blot only before blood ingestion, or at the end of digestion when digested blood is excreted [29]. Since our previous observations show the transcription of *Lltryp2* to be not only constitutive in adult female *L. longipalpis*, but also quite abundant in late larvae and males, it is possible that Lltryp2 protein could be detected in larvae stages or males. We are presently investigating these possibilities.

Zymography has been extensively used to characterize proteolytic activity in crude extracts of many insects, providing information about the relative apparent molecular mass of enzymes responsible for proteolysis. Nevertheless, very few studies are available in sand flies. Using this approach, eleven proteolytic bands from 33 to 102 kDa were detected in *L. longipalpis* L4 larvae [37]. Additionally, up to eleven radio-labeled trypsin or chymotrypsin enzymes were detected in *Lutzomyia anthophora* larvae and adult midguts [38], [39], indicating the presence of isoforms of these proteases in the *Lutzomyia* genus. However, none of these studies examined proteolysis band profile of blood fed adult sandflies. In our studies using gelatin as substrate, we observed that major proteolysis occurred in alkaline conditions (pH 8.0), confirming previous results using females and larvae [37], [40]. We also identified proteolytic bands which appeared and increased in intensity only after blood ingestion, some of them having molecular mass consistent with bands detected by western blot.

Insects might have a complex repertoire of trypsin-like enzymes to digest different blood contents. Indeed the variability of bands we observed when zymography was done using different substrates, suggests the presence of more than one group of enzyme with different specificities. This is consistent with results obtained in *A. gambiae*, where enzyme-substrate specificity was detected as well. Recombinant *Antryp1* digested efficiently hemoglobin and serum albumin, while recombinant *Antryp2* seemed to be active mainly against hemoglobin [28], [29].

The major enzymatic activity detectable in *L. longipalpis* midgut samples may be classified as trypsin-like, since in our zymography results obtained in the presence of class-specific protease inhibitors, only serine protease inhibitors were able to reduce the midgut proteolytic activity, and trypsin specific inhibitors were capable of inhibiting almost completely the activities. Similar zymography results were described in *A. stephensi* [41], where serine proteases were shown to be the most abundant enzymes after blood ingestion. Still, we cannot rule out the presence of some minor chymotrypsin-like protease activities, which have been already identified in *P. papatasi* [36].

Since serine protease inhibition in the phlebotomine midgut was previously associated to *Leishmania* infection [15]–[17], we investigated the effect of *L. i. chagasi* infection on *L. longipalpis* midgut enzymatic activity, by in-solution enzymatic assays using trypsin specific substrate. We observed that the trypsin activity peak is shifted from 12 h to 24 h ABF in infected samples with more evident differences between infected and non-infected guts at 12 and 48 h post blood meal. Also, we observed that total trypsin activity was not completely reduced by the parasite infection, suggesting that more than one trypsin-like enzyme was active in the midgut samples or that infection had a partial reducing effect on trypsin activities. A similar reducing effect was described in *P. papatasi*, where *L. major* infection resulted in a decrease of enzymatic activity during initial stages of digestion, which might help transitional-stage parasite survival in the midgut of the insect [16]. Using a non natural but well established model, Sant'Anna *et al*. [17] also showed that *L. longipalpis* trypsin activity is reduced in the presence of the *L. mexicana* infection from 24 h to 72 h ABF, and the direct inhibition of trypsin gene expression by RNAi led to increased parasite numbers in the midguts.

Serine protease inhibitors (ISP) were found in *L. major*, with inhibitory effect over vertebrate macrophage serine proteases, such as neutrophil elastase, trypsin and chymotrypsin [42]. The authors raised the possibility of ISP having an effect on insect midgut proteases. One possible explanation for the reduced protease activity detected in infected *L. longipalpis* could be the presence of ISP-like molecule produced by *L. i. chagasi*, although so far the presence of such a molecule has not been described in this parasite.

In *A. aegypti*, the early trypsin transcription and synthesis have been shown to be triggered by aminoacids released in the midgut after initial blood digestion, and the Target of Rapamycin (TOR) kinase has been implicated as one participant of such a nutrient sensing mechanism [43]. In *Anopheles aquasalis* there is a large decrease in expression of a serine protease upon infection by *Plasmodium vivax*, indicating a direct effect of the parasite on vector gene regulation (our unpublished data). If such a mechanism occurs in *L. longipalpis*, *L. i. chagasi* would be able to stimulate a reduction of trypsins transcription or synthesis, by producing a molecule capable of interfering in the early trypsin synthesis pathway. This is yet unclear for Lltryp1 and Lltryp2 trypsins. Jochim *et al*. [10] indicated that *Lltryp1* transcripts were less abundant while *Lltryp2* transcripts were more abundant in infected *L. longipalpis* cDNA libraries. Pitaluga *et al*. [11] detected a similar result for *Lltryp2* but not for *Lltryp1* in their analysis of infected and non-infected midgut cDNA libraries. We have shown by RT-PCR that the transcription of these two genes at 72 h ABF, when *Leishmania* promastigotes attach to midgut cells, was not altered when the insect was infected [18]. We are presently investigating levels of transcription at earlier stages of infection. Preliminary western blot experiments performed with antibodies directed against both trypsins did not show a significant difference in protein levels when comparing infected and non-infected insects.
